# Triglyceride Glycemic Index and Triglyceride/HDL Ratio: Can They be Biomarkers for Insulin Resistance in Euthyroid Women with Hashimoto's Thyroiditis, and Are They Related to Thyroid Function Tests?

**DOI:** 10.2174/0118715303399805251024074458

**Published:** 2026-06-09

**Authors:** Seher ÇetinkayaAltuntaş, Hande Özgen

**Affiliations:** 1 Division of Endocrinology and Metabolism, Department of Internal Medicine, Bursa Yuksek Ihtisas Education and Training Hospital, University of Health Sciences, Bursa, Turkey;; 2 Department of Public Health, Bursa Yuksek Ihtisas Education and Training Hospital, University of Health Sciences, Bursa, Turkey

**Keywords:** Triglyceride glycemic, triglyceride/HDL, ratiTriglyceride/HDL Ratio, hashimoto's Thyroiditis, euthyroid, insulin resistance

## Abstract

**Introduction:**

Hashimoto’s thyroiditis (HT) is the most common autoimmune disease. Insulin resistance (IR), independent of thyroid function tests but attributed to thyroid autoantibodies, can be observed in (HT). The most frequently employed method for demonstrating IR is the HOMA-IR index. Recently, the triglyceride-glucose index (TGI) and triglyceride-to-HDL ratio (TG/HDL) have been proposed as novel, practical, and cost-effective indicators of IR. Thus, this study aimed to investigate the relationship between TGI, TG/HDL ratio, and HOMA-IR in euthyroid, normoglycemic women aged 18-45 years with HT.

**Methods:**

This retrospective study was conducted at a tertiary care center and included 108 women with HT and 62 healthy controls. Parameters recorded for all participants included TSH, free thyroxine (fT4), free triiodothyronine (fT3), anti-thyroglobulin antibody (anti-Tg), anti-thyroid peroxidase antibody (anti-TPO), glucose, triglycerides, HDL, LDL, insulin, HOMA-IR, TGI, and TG/HDL ratio.

**Results:**

Compared to the healthy control group, TGI and TG/HDL were significantly elevated in patients with HT, with respective values of 3.67 vs. 3.55 and 1.83 vs. 1.46 (*p*=0.001, *p*=0.005, respectively). Anti-TPO showed a weak positive correlation with HOMA-IR and moderate positive correlations with TGI and TG/HDL (r=0.280, 0.352, and 0.336, respectively). Similarly, anti-Tg correlated positively with HOMA-IR, TGI, and TG/HDL (r=0.274, 0.187, and 0.159, respectively). A moderate, significant positive correlation was found between HOMAIR and both TGI and TG/HDL (r=0.477 and 0.455, respectively). Receiver operating characteristic analysis indicated that TG/HDL (area under the curve (AUC): 0.802, 95% confidence interval (CI): 0.716–0.888, *p*<0.001) and TGI [AUC: 0.824, 95% CI: 0.741–0.907, *p*<0.001) had diagnostic value in predicting elevated HOMA-IR. Furthermore, a TG/HDL ratio of ≥1.99 predicted elevated HOMA-IR with 80.6% sensitivity and 70.1% specificity, while a TGI ≥ 3.68 had 87.1% sensitivity and 67.5% specificity.

**Conclusion:**

Women with euthyroid, normoglycemic HT exhibit increased HOMA-IR. TGI and TG/HDL are novel, cost-effective, accessible biomarkers that can be used to detect IR early in this patient group and facilitate screening across large populations.

## 1 INTRODUCTİON

Hashimoto’s thyroiditis (HT) is the most prevalent autoimmune disease and the leading cause of primary hypothyroidism in iodine-sufficient regions [1]. HT is a chronic inflammatory disorder characterized by infiltration of the thyroid parenchyma by T cells, leading to destruction and fibrosis, with varying degrees of thyroid dysfunction. Although its pathophysiology has not yet been fully elucidated, HT involves a robust immune response mediated by cytokines released by sensitized T cells and antibodies against thyroid antigens, including thyroid peroxidase antibodies (anti-TPO) and thyroglobulin antibodies (anti-Tg) [2].

Insulin resistance (IR) is a widespread global health concern. It plays a critical role in metabolic syndrome (MetS), obesity, atherosclerosis, and cardiovascular diseases. The hyperinsulinemic-euglycemic clamp technique (HECT) is the gold standard for assessing IR, but it is invasive, costly, and impractical for routine use. Therefore, homeostasis model assessment of IR (HOMA-IR) is the most commonly used method in clinical and research settings. While practical and inexpensive, HOMA-IR has limitations, prompting the search for new biomarkers. Among the methods that have gained attention in this area are the triglyceride glycemic index (TGI) and the triglyceride-to-high-density lipoprotein (TG/HDL) ratio, which have been particularly accepted as reliable indicators of IR in recent years [3]. It has been reported that these measures have a high level of correlation with HECT and have superior efficacy compared to HOMA-IR [4-7].

In this study, we aimed to compare TGI and TG/HDL in normoglycemic, euthyroid women with HT against healthy controls and investigate their correlation with HOMA-IR. Based on a review of existing literature, we hypothesize that even in euthyroid states, patients with HT would exhibit higher HOMA-IR, TGI, and TG/HDL levels than healthy controls.

## 2 MATERIALS AND METHODS

### 2.1 Study Sample

This retrospective, single-center, case-control study was conducted with patients diagnosed with HT who presented to the endocrinology and metabolism outpatient clinic at University of Health Sciences Bursa Yuksek Ihtisas Hospital between January 2023 and November 2024. Data from 363 patients were reviewed, and 255 patients were excluded for not meeting the study criteria described below. The final sample consisted of 108 patients. Data from 62 age- and body mass index (BMI)-matched healthy female controls were retrospectively retrieved from occupational health clinics and routine check-up screenings. All information was extracted from the hospital’s computerized database.

## 2.2 Study Protocol

The study included women aged 18–45 years who were under follow-up for HT. Exclusion criteria were as follows: 1) pregnancy or breastfeeding, 2) TSH levels outside the reference range of 0.35–4.94 µIU/mL, 3) a known diagnosis of type 1 or type 2 diabetes mellitus (T1DM or T2DM), 4) acute or chronic liver or kidney disease, 5) a history of malignancy, and 6) not using a lipid-lowering drug. Sociodemographic characteristics, such as age, gender, marital status, alcohol use, and smoking habits, as well as levothyroxine (LT4) dosage (mcg/day) and duration of use (years), were recorded. Anthropometric measurements, including body weight (kg), height (cm), and waist circumference (cm), were documented, and BMI was calculated as kg/m^2^.

TSH, free thyroxine (fT4), free triiodothyronine (fT3), anti-Tg, and anti-TPO were recorded for both the patient and control groups. The reference ranges for these parameters in our laboratory were as follows: TSH, 0.35–4.94 µIU/mL; fT4, 0.7–1.48 ng/dL; fT3, 1.58–3.91 pg/mL; anti-Tg, 0–4.11 IU/mL; and anti-TPO, 0–5.61 IU/mL Since antibody positivity can occur in normal individuals, titers >45 IU/mL were considered positive. In addition, plasma fasting glucose, triglycerides, HDL, LDL, and insulin levels were recorded for all participants. HOMA-IR was calculated using the following formula: fasting plasma insulin (mIU/L) × glucose (mg/dL) / 405. TGI was calculated using the following formula: Ln (fasting triglycerides (mg/dL) × fasting glucose (mg/dL) / 2). The HOMA-IR value cut-off value was 2.5. The TG/HDL ratio was calculated by dividing triglycerides by HDL cholesterol [3, 8]. All data were compared with the healthy control group.

Before the study, approval was obtained from the Ethics Committee of Bursa Yuksek Ihtisas Training and Research Hospital (approval number: 2024-TBEK 2024/12-9, date:18/12/2024). All procedures in the study adhered to the ethical standards of the institutional and/or national research committee and the 1964 Declaration of Helsinki and its later amendments or comparable ethical standards.

### 2.3 Statistical Analysis

Statistical analyses were performed using SPSS version 25. Descriptive findings for categorical variables were expressed as counts and percentages, while continuous variables were presented as mean ± standard deviation or median (minimum–maximum) based on normality. The Kolmogorov-Smirnov test was used to assess the distribution of continuous variables. For binary analyses, the chi-square test was used for categorical variables, and the t-test or Mann-Whitney U test was used for continuous variables, depending on normality. Correlation coefficients and statistical significance were calculated using the Spearman test for non-normally distributed variables. Receiver operating characteristic (ROC) analysis was employed to evaluate the role of TG/HDL and TGI in predicting elevated HOMA-IR in the case group. Statistical significance was set at *p* < 0.05.

## 3 RESULTS

The study included 170 female participants with a mean age of 40.15 ± 9.45 years and a mean BMI of 27.03 ± 4.73. Of the participants, 29.4% (n = 50) were postmenopausal, and 71.8% (n = 122) had never smoked. There were no significant differences between the two groups in terms of age, BMI, marital status, menopause status, smoking, or alcohol use [*p* ≥ 0.0^5^]. Sociodemographic characteristics by group are presented in Table **1**.

Among women with HT, 53.7% (n = 58) were using LT4, with a median duration of 6 (range: 1–30) years and a median dose of 100 (range: 20–200) mcg/day. Compared to the control group, women with HT had significantly higher levels of TSH, anti-TPO, anti-Tg, glucose, LDL, triglycerides, HOMA-IR, TGI, and TG/HDL, while free T3 levels were significantly lower. Laboratory values for women with HT and the control group are shown in Table **2**.

Across the entire study group, anti-TPO showed a weak positive correlation with HOMA-IR and a moderate positive correlation with TGI and TG/HDL (rho = 0.280, 0.352, and 0.336, respectively). Anti-Tg demonstrated weak positive correlations with HOMA-IR, TGI, and TG/HDL (rho = 0.274, 0.187, and 0.159, respectively).

A moderate, significant positive correlation was observed between HOMA-IR and both TGI and TG/HDL (Spearman’s rho = 0.477 and 0.455, respectively, *p* < 0.01). Among all participants, 19.2% (n = 33) had HOMAIR values above the normal range. In the case group, 28.7% (n = 31) exceeded the normal HOMA-IR threshold, compared to only 3.2% (n = 2) in the control group, with a significant difference between the groups (*p* ≤ 0.01). No correlation was observed between TSH, free T4, and TGI or TG/HDL.

ROC analysis demonstrated that in women with HT, TG/HDL (area under the curve (AUC): 0.802, 95% confidence interval (CI): 0.716–0.888, *p* < 0.001) and TGI (AUC: 0.824, 95% CI: 0.741–0.907, *p* < 0.001) had diagnostic value in predicting elevated HOMA-IR Fig. (**1**). According to the analysis, a TG/HDL ratio of ≥1.99 predicted elevated HOMA-IR with 80.6% sensitivity and 70.1% specificity, while a TGI of ≥3.68 predicted elevated HOMA-IR with 87.1% sensitivity and 67.5% specificity in euthyroid women with HT. Data are presented in Table **3**.

## 4 DISCUSSION

This study is the first to compare TGI and TG/HDL between euthyroid, normoglycemic women with HT and healthy women aged 18–45 years. According to the findings, women with euthyroid HT exhibited higher TGI and TG/HDL levels compared to healthy individuals. These elevated levels were correlated with anti-Tg and anti-TPO. The study demonstrated that TGI and TG/HDL could be utilized to predict IR.

Studies have shown that TGI and TG/HDL are correlated with HECT, the gold standard for identifying IR [9]. However, HECT is labor-intensive, costly, and impractical for routine application. Therefore, there is ongoing research to identify sensitive, practical, accurate, and simple methods for detecting IR. Currently, the most commonly used method is HOMA-IR. However, this method also has certain disadvantages. For example, in normoglycemic or underweight patients, HOMA-IR may yield false negatives; its reproducibility across repeated tests is challenging, and it requires insulin measurement [10]. In contrast, measurements of TGI and TG/HDL are inexpensive, simple, and reliable, making them suitable for both clinical use and research [11]. One of the most significant advantages is their ability to detect IR at earlier stages, as supported by other studies showing that these markers can identify IR even in normoglycemic, non-obese individuals and entirely healthy individuals [12-15]. Our study also supports this finding, revealing a mean BMI of 27.03 ± 4.73. Consistent with this, TGI and TG/HDL in our study predicted elevated HOMA-IR values. While they may not surpass HOMA-IR, they serve as a viable alternative in situations where insulin measurements are unavailable.

Under normal conditions, insulin physiologically inhibits the release of free fatty acids from adipose tissue and stimulates TG storage in adipocytes. In cases of IR, this mechanism is disrupted, leading to increased lipolysis and the release of free fatty acids into circulation. This results in hypertriglyceridemia and elevated very-low-density lipoprotein levels. Another mechanism involves increased hepatic lipase activity in IR, which accelerates HDL catabolism, resulting in smaller, unstable HDL particles. These lipid profile alterations manifest as increased TG levels and decreased HDL levels. TGI and TG/HDL serve as indirect indicators of IR due to this mechanism.

Although contrary findings exist, the presence of autoimmune thyroid disease has been suggested to be an independent factor for cardiovascular disease (CVD), obesity, MetS, and IR, regardless of thyroid function tests [16-19]. HT should not be classified as merely an organ-specific autoimmune disease, as extrathyroidal findings are common in these patients. These systemic manifestations are attributed more to thyroid autoantibodies (particularly anti-TPO) than thyroid function tests (TFTs) [20]. These antibodies are implicated in chronic inflammation, immunological pathways, and endothelial dysfunction [21]. Especially in HT patients, early detection of atheroma plaques has been identified as a risk factor for CVD. Antibodies have been held responsible for this. A study by Lei *et al*. found no differences in lipid and glucose parameters between euthyroid patients with HT and a control group; however, HOMA-IR values were higher in those with HT. In groups with elevated TSH levels, both immunological markers and lipid-glucose metabolism indicators were worse (hypothyroid HT >euthyroid HT > control). These results suggest that metabolic parameters and the severity of immunological markers, reflected clinically by elevated anti-Tg and anti-TPO, are directly proportional to TSH levels [21]. In contrast, some studies have found no association between thyroid autoantibodies and IR, obesity, or MetS [16, 22, 23]. A common aspect of these studies is that they all used HOMA-IR as the method for calculating IR. To date, no studies have examined TGI and TG/HDL levels in patients with HT, making this research novel.

This study revealed that impaired metabolic parameters and IR in patients with HT were associated with thyroid antibody levels. Another potential explanation could be related to the TFTs of these patients. Although no relationship was identified between TFTs and TGI or TG/HDL in this study, we hypothesize that normal-high TSH and normal-low free T3 in euthyroid HT women might contribute to metabolic issues. The detected TFTs might exacerbate metabolic problems, as previous research has shown that such TSH and free T3 levels contribute to obesity, dyslipidemia, and IR [24]. However, certain questions remain unresolved, including whether specific TSH reference ranges should be tailored for HT patients and whether early LT4 therapy should be administered to lower high-normal TSH levels.

No definitive cut-off values for TGI and TG/HDL levels have been established. For TGI, a cut-off of 3.23 has been proposed, while our study suggests 3.68 [25]. For TG/HDL, cut-offs of >2.5 for women and <2.8 for men have been suggested in African and American populations, while cut-offs of 1.5 for women and 2 for men have been proposed for other populations [6]. Our study identified 1.9 as the cut-off for TG/HDL. These findings warrant further investigation in additional studies.

The strengths of this study include its focus on TGI and TG/HDL in euthyroid, normoglycemic, non-morbidly obese HT women, as well as the novel exploration of their relationship with IR. The lack of menopausal differences between the case and control groups partially mitigates the confounding metabolic effects of menopause. The limitations of the study are its retrospective design, lack of thyroid ultrasound data for patients with HT, lack of data on physical activity, dietary patterns, body fat distribution, and the small sample size. Interpretation of the results would have been more meaningful in the presence of this information. In the presence of patients with an autoimmune pattern on thyroid ultrasound, a more accurate diagnosis of autoimmune thyroid disease would have been made.

## CONCLUSION

Even euthyroid women with HT exhibit elevated IR and carry metabolic and cardiovascular risks. TGI and TG/HDL are promising new methods for detecting IR in patients with HT. These markers are inexpensive, accurate, and accessible, making them suitable for early-stage detection and large-scale population screening. Moreover, in euthyroid patients, interventions aimed at maintaining TSH at the lower end of the reference range or developing therapies targeting thyroid autoantibody levels may help reduce these risks. Further comprehensive research is warranted to confirm and expand upon these findings.

## Figures and Tables

**Fig. (1):**
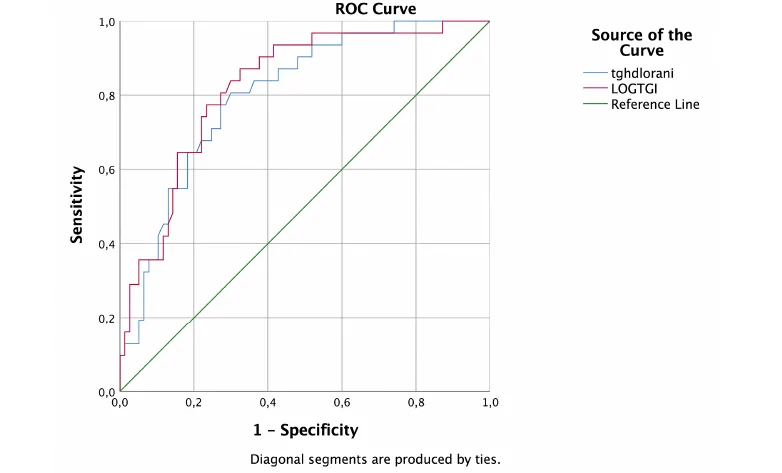
ROC curve evaluating the role of TG/HDL and TGI in predicting elevated HOMA-IR in patients with Hashimoto’s thyroiditis. The **red line** represents the TyG index (AUC = 0.824; 95% CI: 0.741–0.907; *p* < 0.001). The **blue line** represents the TG/HDL ratio (AUC = 0.802; 95% CI: 0.716–0.888; *p* < 0.001). The **green diagonal line** shows the reference line of no discrimination (AUC = 0.5).

**Table 1:** Sociodemographic characteristics of patients with Hashimoto’s thyroiditis and healthy controls.

-		**Hashimoto’s Thyroiditis (n = 108)**	**Healthy Control ** **(n = 62)**	***p*-value**
**Age (mean ± SD) (years) **		40.81 ± 9.39	39.02 ± 9.52	0.236
**BMI (mean ± SD)**		27.44 ± 5.10	26.31 ± 3.96	0.109
**Marital status**	**Married, % (n)**	13.0 (14)	%24.2% (15)	0.061
	**Single, % (n)**	87.0% (94)	75.8% (47)	
**Menopause**	**Yes, % (n)**	33.3% (36)	22.6% (14)	0.139
	**No, % (n)**	66.7% (72)	77.4% (48)	
**Smoking status**	**Active smoker, % (n)**	21.3% (23)	16.1% (10)	0.661
	**Ex-smoker, % (n)**	9.3 (10)	8.1% (5)	
	**Never-smoker, % (n)**	69.4% (75)	75.8% (47)	
**Alcohol consumption**	**Current drinker, % (n)**	3.7% (4)	6.5% (4)	0.197
	**Former drinker, % (n)**	2.8% (3)	8.1% (5)	
	**Never consumed,** ** % (n)**	93.5% (101)	85.5% (53)	

**Abbreviations:** SD: standard deviation, BMI: body mass index.

**Table 2:** Comparison of laboratory values between patients with Hashimoto’s thyroiditis and healthy controls.

-	**Hashimoto’s Thyroiditis (n=108)**	**Healthy Control ** **(n = 62)**	***p*-value**
**TSH (mean ± SD)**	2.40 ± 1.47	1.67 ± 1.00	**0.001**
**Serbest T4 (mean ± SD)**	1.01 ± 0.22	0.98 ± 0.12	0.207
**Serbest T3 (mean ± SD)**	2.72 ± 0.44	2.87 ± 0.42	**0.026**
**Anti-TPO (median, min-max)**	340.41 (0.51–1000.00)	0.54 (0.00–37.85)	**0.000**
**Anti-Tg (median, min-max)**	48.04 (0.10–1000.00)	0.94 (0.20–22.12)	**0.000**
**Fasting blood glucose (mean ± SD)**	91.79 ± 12.18	86.55 ± 6.61	**0.000**
**LDL (mean ± SD)**	118.40 ± 35.32	107.14 ± 28.68	**0.034**
**Total cholesterol (mean ± SD)**	195.18 ± 44.91	183.35 ± 34.73	0.075
**HDL (mean ± SD)**	55.78 ± 14.61	59.49 ± 11.43	0.068
**Triglyceride (mean ± SD)**	117.18 ± 66.93	89.29 ± 40.75	**0.001**
**HbA1C (mean ± SD)**	5.28 ± 0.50	5.18 ± 0.42	0.157
**Insulin (mean ± SD)**	9.38 ± 5.18	6.89 ± 2.56	**0.000**
**HOMA-IR (median, min-max)**	1.93 (0.64–6.83)	1.41 (0.54–3.35)	**0.001**
**TGI (mean ± SD)**	3.67 ± 0.24	3.55 ± 0.20	**0.001**
**Triglyceride/HDL ratio (median, min–max)**	1.83 (0.37–9.45)	1.46 (0.17–6.46)	**0.005**

**Abbreviations:** TSH: thyroid-stimulating hormone, HOMA-IR: homeostasis model assessment of insulin resistance, anti-TPO: thyroid peroxidase antibody, anti-Tg: thyroglobulin antibody, TGI: triglyceride glycemic index, HDL: high-density lipoprotein, LDL: low-density lipoprotein, HbA1C: glycosylated hemoglobin.

**Table 3:** Sensitivity, specificity, accuracy, and predictive values of TG/HDL and TGI in differentiating patients with Hashimoto’s thyroiditis from healthy controls.

-	Cut-off	Sensitivity %	Specificity%	Accuracy%	Positive Predictive Value %	Negative Predictive Value %
TG/HDL	1.50	87.1	54.5	28.7	43.5	91.3
	1.99	80.6	70.1	71.3	50.0	89.7
TGI	3,23	100.0	5.2	32.4	29.8	100.0
	3,68	87.1	67.5	73.1	51.8	92.9

**Abbreviations: **TG: triglyceride, HDL: high-density lipoprotein, TGI: triglyceride glycemic index.

## Data Availability

The data and supportive information are available within the article.

## LIST OF ABBREVIATIONS

HT: Hashimoto’s Thyroiditis
IR: Insulin Resistance
TGI: Triglyceride-Glucose Index
fT4: Free Thyroxine
fT3: Free Triiodothyronine
anti-Tg: Anti-Thyroglobulin Antibody
anti-TPO: Anti-Thyroid Peroxidase Antibody
MetS: Metabolic Syndrome
HECT: Hyperinsulinemic-Euglycemic Clamp Technique
ROC: Receiver Operating Characteristic
CVD: Cardiovascular Disease
